# Is Bridge Plating of Comminuted Humeral Shaft Fractures Advantageous When Using Compression Plates with Three versus Two Screws per Fragment? A Biomechanical Cadaveric Study

**DOI:** 10.1155/2021/6649712

**Published:** 2021-03-06

**Authors:** Guilherme Seva Gomes, Ivan Zderic, Marc-Daniel Ahrend, Kodi E. Kojima, Peter Varga, William Dias Belangero, Geoff Richards, Simon M. Lambert, Boyko Gueorguiev

**Affiliations:** ^1^AO Research Institute Davos, Davos, Switzerland; ^2^Department of Traumatology and Reconstructive Surgery, BG Trauma Center Tübingen, Eberhard-Karls University of Tübingen, Tübingen, Germany; ^3^Institute of Orthopedics and Traumatology, University of Sao Paulo, Brazil; ^4^Department of Orthopedics and Traumatology, Faculty of Medical Sciences, State University of Campinas, Sao Paulo, Brazil; ^5^Department of Trauma and Orthopaedics, University College London Hospital, London, UK

## Abstract

**Background:**

Minimally invasive plate osteosynthesis (MIPO) is one of the generally accepted surgical techniques for the treatment of humeral shaft fractures. However, despite the high bone union rate, a variety of complications are still prevailing. Moreover, the current literature lacks data comparing the anterolateral MIPO approach using dynamic compression plates accommodating different numbers of screws. The aim of this study was to analyze the biomechanical performance of comminuted humeral shaft fractures fixed with dynamic compression plates using either two or three screws per fragment.

**Methods:**

Six pairs of fresh-frozen human cadaveric humeri from donors aged 66.8 ± 5.2 years were randomized to two paired study groups for simulation of bridge-plated comminuted shaft fracture type AO/OTA 12-C1/2/3 without interfragmentary bony support, using a dynamic compression plate positioned on the anterolateral surface and fixed with two (group 1) or three (group 2) screws per fragment. All specimens underwent nondestructive quasistatic biomechanical testing under lateral bending, anterior bending, axial bending, and torsion in internal rotation, followed by progressively increasing cyclic torsional loading in internal rotation until failure.

**Results:**

Initial stiffness of the plated specimens in lateral bending, anterior bending, axial bending, and torsion was not significantly different between the groups (*P* ≥ 0.22). However, cycles to 10°, 15°, and 20° torsional deformation and cycles to construct failure were significantly higher in group 2 compared with group 1 (*P* ≤ 0.03).

**Conclusions:**

From a biomechanical perspective, no significant superiority is identified in terms of primary stability when using two or three screws per fragment for bridge compression plating of comminuted humeral shaft fractures. However, three-screw configurations provide better secondary stability and maintain it with a higher resistance towards loss of reduction under dynamic loading. Therefore, the use of a third screw may be justified when such better secondary stability is required.

## 1. Introduction

Anterior, lateral, or posterior dynamic compression plate fixation, bridge plating, and intramedullary nailing represent the generally accepted surgical techniques for diaphyseal humeral fracture treatment [1–3]. Fixation with either locking plates or dynamic compression plates is considered the treatment of choice [3, 4]. From a biomechanical perspective, plate fixation offers an advantage of providing higher torsional stability when compared with intramedullary nailing [5, 6]. This advantage becomes particularly effective at the humerus where torsional moments dominate over axial forces, in contrast to other long bones, such as in the lower extremity region [7, 8]. Prevention of excessive interfragmentary shear movements, induced by torsional loading, has been reported to positively trigger the bone healing process [9]. Moreover, locked plating is considered advantageous over conventional compression plate fixation in case of poor bone quality, featuring enhanced resistance against screw pullout and cutout [10–13]. However, in the case of normal bone quality, this advantage remains debatable [14]. Despite the high bone union rate following surgical treatment by plating [15], a variety of complications is still prevailing, including late consolidation, infection, shoulder disability, and postoperative paralysis [3, 15].

A more biological approach with less soft tissue irritation was realized with the introduction of the minimally invasive plate osteosynthesis (MIPO) as an alternative to standard bridge plating of comminuted humeral shaft fractures [2]. The plate is applied on the anterolateral humeral shaft surface and fixed with two to four screws per fragment. Effectiveness, safety of application, and good outcomes have been demonstrated from clinical, anatomical, and radiologic perspectives [1, 2, 16–19]. The advantages over standard plating techniques include shorter operative time and less blood loss. Using a long narrow dynamic compression plate with two 4.5 mm cortical screws per fragment was initially recommended for plating of comminuted humeral fractures [1, 2]. Meanwhile, the assortment of implants applicable for this technique has been extended to include broad plates and locking compression plates (LCPs), considering insertion of up to six screws per fragment [2, 18–20].

Although the biomechanics of humeral shaft fracture fixation has been extensively investigated, the current literature surprisingly lacks data comparing the anterolateral MIPO approach with bone-implant constructs using dynamic compression plates accommodating different numbers of screws [14, 21–26]. Therefore, to expand the understanding of anterolateral plating, the aim of this study was to analyze the biomechanical performance of comminuted humeral shaft fractures bridged with a dynamic compression plate using either two or three screws per fragment. Relying on the findings of the previous work comparing the same screw constellations with the use of locking plates [24], it was hypothesized that no significant differences between dynamic compression plating with two and three screws per fragment would be observed.

## 2. Materials and Methods

### 2.1. Specimens and Preparation

Six pairs of fresh-frozen (–20°C) human cadaveric humeri from donors aged 66.8 ± 5.2 years (mean ± standard deviation) (range 61–77 years) were used in this study. Soft tissue was removed, and radiographs were obtained to exclude any abnormal morphologies or lesions. Bone mineral density (BMD) was accessed by means of high-resolution peripheral computed tomography (XtremeCT, SCANCO Medical AG, Brüttisellen, Switzerland). Scanning was performed at a resolution of 82 *μ*m within the proximal 120 mm measured from the top of the humeral head. The region of interest for BMD evaluation in the humeral head reached from the lower margin of the anatomical neck 25 mm proximally. All specimens were thawed at room temperature 24 hours prior to preparation and biomechanical testing, and kept moist by wrapping in saline-soaked gauzes. The proximal and distal 35 mm bone ends were embedded in polymethylmethacrylate (PMMA, SCS-Beracryl D28, Suter Kunststoffe AG, Fraubrunnen, Switzerland) cylindrical forms. During embedding, care was taken to align the specimen's anatomical axis—defined as the straight line connecting the glenohumeral joint center with the center point between the medial and lateral epicondyles at the elbow [27]—with the axes of both embedding cylindric forms.

**Figure 1:**
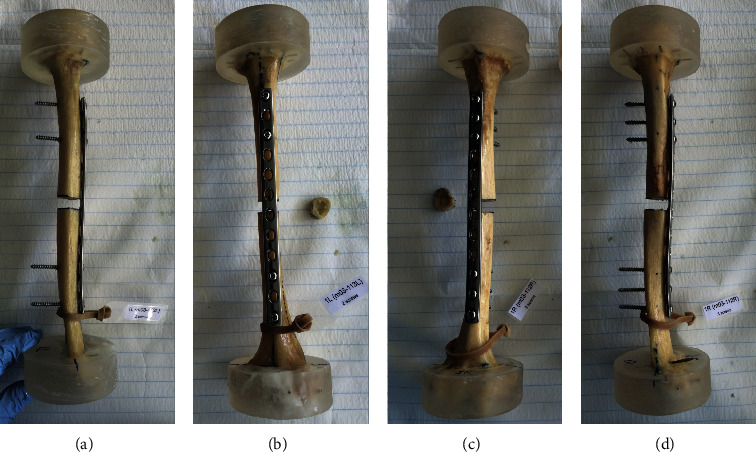
Photographs of two paired specimens plated with LC-DCP on the anterolateral side of the humerus and fixed with either two (a, b) or three (c, d) screws per fragment.

The humeri were randomly assigned to two paired study groups for bridge plating using a total of four (group 1) or six (group 2) screws per specimen. Instrumentation was performed by the same surgeon in the intact state of the specimens using a narrow stainless-steel (316L) 214 mm long, 12 mm wide, and 4 mm thick 12-hole 4.5 mm Limited Contact Dynamic Compression Plate (LC-DCP, DePuy Synthes, Zuchwil, Switzerland) featuring hole space distances of 18 mm. Cortical 4.5 mm screws were used for bicortical fixation. Each plate was preshaped to fit well on the anterolateral humerus surface in central position over the diaphysis with temporary fixation by two forceps. To mimic the clinical scenario as prescribed in a previous study [2], the most proximal (#1) and most distal (#12) plate holes were occupied first, followed by the two closest holes located next to the marked osteotomy site (#3 and #10, Figure 1). A 3.2 mm drill bit was used together with an LC-DCP drill guide for pilot holes, followed by pretapping with a 4.5 mm tap. Subsequently, all screws were inserted with a standard screwdriver. This procedure reflected the four-screw configuration in group 1. For the instrumentation in group 2, plate holes #2 and #11 were additionally occupied with screws. As a result, the working length in both groups was standardized to 126 mm. Following instrumentation, a 10 mm transverse osteotomy gap was created in the middle of the diaphysis using a standard oscillating saw to simulate a comminuted AO/OTA 12-C1/2/3 fracture without interfragmentary bony support. Care was taken to avoid damage of the plate surface. All screws were retightened after osteotomy setting.

The remaining bone slice from the osteotomy procedure was used to measure the cortical thickness at the anterior and posterior diaphyseal sides with a caliper. The applied compressive force with the caliper was high enough to disrupt the thin trabecular bone on the endosteal surface and get in contact with the inner cortical wall. The measurements were repeated twice for each specimen, and their average among both diaphyseal sides was defined as the cortical average and used for correlation analysis.

### 2.2. Biomechanical Testing

**Figure 2:**
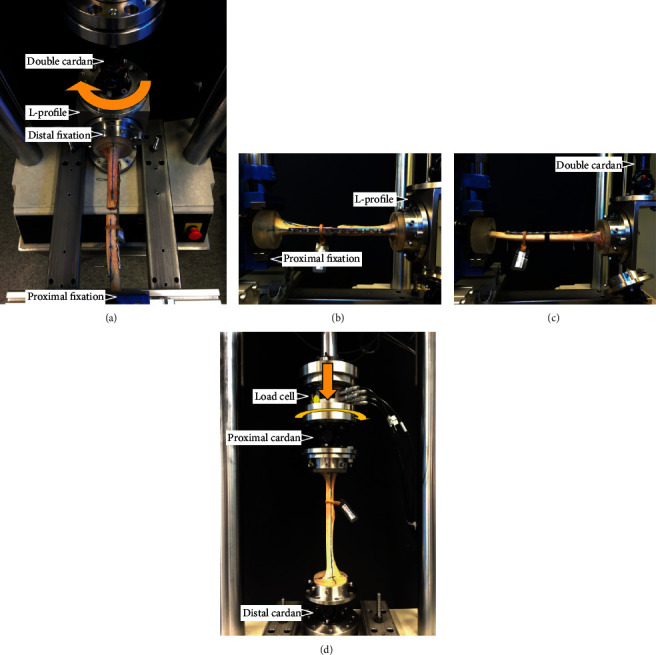
Setup with a specimen mounted for biomechanical testing in lateral bending (a, b), anterior bending (c), axial bending, and torsion in internal rotation (d). Arrows in (a) and (d) denote loading directions.

Biomechanical testing was performed on a biaxial servo-hydraulic material testing system (Mini Bionix II 858, MTS Systems Corp., Eden Prairie, MN, USA) equipped with a 4 kN/100 Nm load cell. First, each humerus underwent four consecutive nondestructive quasistatic tests in its intact state as follows: (1) lateral bending, simulating abduction induced by the deltoid and rotator cuff in the proximal fragment; (2) anterior bending, simulating the forces acting at the anterior compartment during any weight lifting or elbow flexing movement; (3) axial bending, simulating resisted elbow extension; (4) torsion in internal rotation, simulating rotation of the upper extremity introduced by the external rotators at the proximal fragment. After plating and fracture creation, all four nondestructive tests were repeated to mimic the forces and moments acting at the humerus during the initial rehabilitation period after surgery, followed by a destructive cyclic torsional test in internal rotation. The setup for each separate test was adapted from previous works (Figure 2) [28, 29].

For the lateral and anterior bending tests (Figures 2(a)–2(c)), each specimen was oriented horizontally with its proximal embedding rigidly fixed to the machine base. An L-shaped metal profile composed of two perpendicular base plates was used to connect the distal part of the specimen's embedding to the machine actuator via a double cardan joint. The cardan joint's driving shaft (connected to the machine actuator), the intermediate shaft, and the driven shaft (attached to the L-profile) were aligned with the machine actuator axis. With this assembly constellation, the cardan's rotational axis and the humeral shaft axis intersected each other at a 90° angle, allowing transformation of the generated actuator torque to pure bending moments acting on the humerus. The horizontally oriented specimens were kept free from shear stresses by maintaining the axial force along the actuator axis at 0 N. Furthermore, any potential axial stresses were compensated by the inherent kinematic characteristics of the double cardan joint, while potential torsional moments were eliminated based on the intersection of the two mechanical axes. The machine actuator's rotational capabilities were exploited to initiate three torsional ramped unidirectional loading cycles from 0 Nm to 3 Nm at a rate of 6 Nm/min, being transmitted to stress the specimens with equivalent magnitude in pure bending.

For the axial bending test (Figure 2(d)), each specimen was oriented vertically, aligned with the machine axis, and connected to the machine base and actuator via a single cardan joint on each side. Axial loading was introduced via the actuator in three ramped cycles from 0 N to 100 N at a rate of 200 N/min, while keeping 0 Nm torque.

Both nondestructive quasistatic and destructive cyclic torsional tests were performed using the setup for axial bending. During the nondestructive test in internal rotation, the uniaxial torque was applied in three ramped cycles from 0 Nm to 2 Nm at a rate of 4 Nm/min, while keeping the axial load at 0 N. The destructive cyclic internal rotation test was initiated with a ramp from 0 Nm to 0.5 Nm at a rate of 0.1 Nm/s, followed by progressively increasing sinusoidal torsional loading at 1 Hz, keeping the axial load at 0 N during the whole test. While the valley torque of each cycle was kept constant at 0.5 Nm, the peak torque, starting at 1 Nm, increased at a rate of 0.002 Nm/cycle. The test stop criterion was defined as reaching a 90° angle by the machine actuator with respect to its position at the test start, being found sufficient for retrospective failure analysis.

### 2.3. Data Acquisition and Statistical Analysis

Time (s), axial displacement (mm), and load (N), as well as torsional angle (°) and torque (Nm), were recorded from the machine controllers at 128 Hz.

Each of the nondestructive quasistatic tests served to determine the respective specimen's stiffness, derived from the ascending slope of the axial load-displacement or torsional torque-angle curve in its quasilinear elastic region in the range of 1–3 Nm for pure lateral or anterior bending, 50–100 N for axial bending, and 1–2 Nm for torsion in internal rotation. The stiffness of the plated specimens was normalized versus their intact state.

Furthermore, relative bending and torsional stiffness of the bone-implant constructs were calculated and compared with the corresponding theoretical relative stiffness of the isolated plate. According to the Euler-Bernoulli beam theory [30], the relative bending and torsional stiffness of the isolated plate was calculated as *E* *I*_b_ and *G* *I*_t_, respectively, where *E* is Young's modulus, *I*_b_ is the area moment of inertia, *G* is the shear modulus, and *I*_t_ is the torsional moment of inertia. Further, the relative bending and torsional stiffness of the bone-implant constructs were calculated as *M*_B_*L*/2*α*  and *M*_T_ *L*/*α*, respectively, where *L* is the construct spanning length, *α* is the angular displacement of the machine transducer, *M*_B_ is the applied bending moment, and *M*_T_ is the applied torsional moment. All relative stiffness calculations were related to a unit length under consideration of the following parameter values: *E* = 190 GPa, *G* = 77 GPa, *I*_b_ = 64 mm^4^ for lateral bending, *I*_b_ = 576 mm^4^ for anterior bending, *I*_t_ = 202 mm^4^, *L* = 350 mm, *M*_B_ = 3 Nm, *M*_T_ = 2 Nm, *α* ∈ [1°–3°] for both lateral and anterior bending loading, and *α* ∈ [3°–4°] for torsional loading.

The data from the cyclic test was analyzed with regard to torsional deformation (°) at valley torque after 500, 1000, 1500, 2000, 2500, 3000, and 3500 cycles. In addition, the numbers of cycles until 5°, 10°, 15°, and 20° torsional deformation at valley torque were derived together with the corresponding peak torque values of those cycle numbers. Finally, the number of cycles to failure was determined at the time point when an abrupt change in the torsional angle-time curve was detected. All calculations were performed in valley loading condition (0.5 Nm torque) to consider only plastic deformations of the bone-implant constructs.

Statistical analysis was performed using the IBM SPSS software package (version 27, IBM SPSS Statistics, Armonk, NY, USA). Normal data distribution was screened and proven with the Shapiro-Wilk test. The paired-sample *t*-test was used to detect significant differences between the two groups. General linear model repeated measures test was conducted to screen the progression of both (1) torsional deformation over predefined numbers of cycles and (2) number of cycles until predefined torsional deformation levels. Level of significance was set to 0.05 for all statistical tests.

## 3. Results

An overview of the results from both quasistatic and cyclic tests is presented in Table 1.

### 3.1. Nondestructive Quasistatic Tests

Stiffness of the intact humeri revealed no significant differences between the groups for lateral bending (group 1: 3.7 ± 1.0 Nm/°; group 2: 5.0 ± 1.1 Nm/°), anterior bending (group 1: 4.0 ± 1.0 Nm/°; group 2: 5.3 ± 1.4 Nm/°), axial bending (group 1: 1669.3 ± 200.4 N/mm; group 2: 1864.4 ± 298.9 N/mm), and torsion in internal rotation (group 1: 3.0 ± 0.9 Nm/°; group 2: 3.5 ± 0.3 Nm/°) (*P* ≥ 0.11).

Similarly, stiffness of the plated specimens in lateral bending (group 1: 2.8 ± 1.4 Nm/°; group 2: 3.1 ± 1.2 Nm/°), anterior bending (group 1: 1.4 ± 0.2 Nm/°; group 2: 1.5 ± 0.2 Nm/°), axial bending (group 1: 387.5 ± 143.3 N/mm; group 2: 394.4 ± 134.3 N/mm), and torsion in internal rotation (group 1: 0.7 ± 0.2 Nm/°; group 2: 0.8 ± 0.1 Nm/°) was not significantly different between the groups (*P* ≥ 0.22).

Moreover, normalized stiffness in group 1 and group 2 was 85.1 ± 59.5% and 61.5 ± 18.1% for lateral bending, 35.8 ± 9.3% and 31.6 ± 13.2% for anterior bending, 23.1 ± 7.7% and 21.2 ± 6.3% for axial bending, and 23.8 ± 4.4% and 8 for torsion, respectively, with no significant differences between the groups (*P* ≥ 0.42).

Relative anterior and lateral bending stiffness among all bone-implant constructs ranged between 10.0 Nm^2^ and 30.1 Nm^2^, whereas for the isolated plate, the relative stiffness was 12.2 Nm^2^ for lateral bending and 109.4 Nm^2^ for anterior bending. Relative torsional stiffness of the bone-implant constructs ranged between 10.0 Nm^2^ and 13.7 Nm^2^, while it was 15.6 Nm^2^ for the isolated plate.

### 3.2. Destructive Cyclic Torsional Test

**Figure 3:**
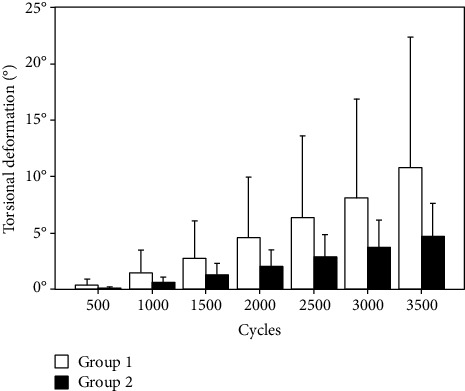
Bar diagram presenting the torsional deformation after 500, 1000, 1500, 2000, 2500, 3000, and 3500 cycles in the two study groups in terms of mean value and standard deviation.

**Figure 4:**
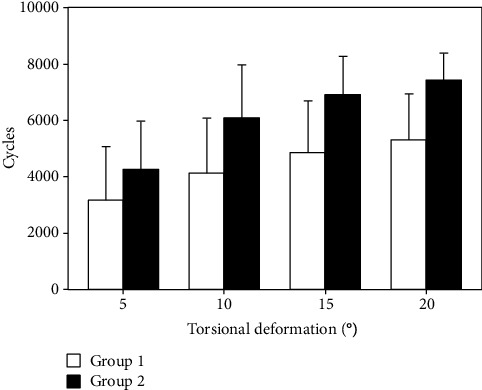
Bar diagram presenting the numbers of cycles and corresponding peak torque until 5°, 10°, 15°, and 20° torsional deformation in the two study groups in terms of mean value and standard deviation. Stars indicate significant differences.

**Figure 5:**
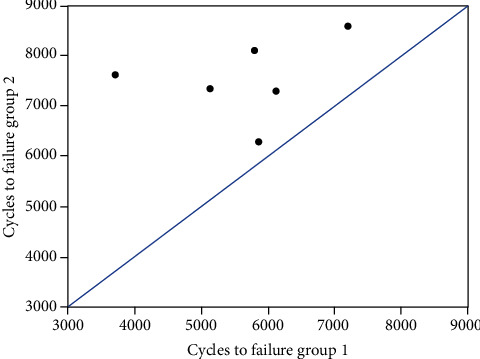
Scatter plot presenting the number of cycles to failure in the two study groups. Each specimen pair is represented by one dot whose *x*- and *y*-coordinates reflect the number of cycles to failure of group 1 and group 2, respectively.

Torsional deformation increased significantly between 500, 1000, 1500, 2000, 2500, 3000, and 3500 cycles in each one of the groups (*P* < 0.01) (Figure 3). Although it was smaller in group 2 compared with group 1, the differences between the two groups remained nonsignificant after 500, 1000, 1500, 2000, 2500, 3000, and 3500 cycles (*P* ≥ 0.22). In addition, both numbers of cycles and corresponding peak torque values until 5°, 10°, 15°, and 20° torsional deformation increased significantly between those angular levels (*P* < 0.01) (Figure 4). For each of these torsional deformation levels, group 2 revealed higher number of cycles and corresponding peak torque value as compared with group 1, being significant for 10°, 15°, and 20° (*P* ≤ 0.03) but not for 5° (*P* = 0.24). BMD (group 1: 193.3 ± 49.0 mg HA/cm^3^, group 2: 205.3 ± 33.0 mg HA/cm^3^) and cortical average (group 1: 4.35 ± 1.66 mm, group 2: 4.98 ± 1.15 mm) were well randomized between the groups (*P* ≥ 0.34) and revealed no significant effect as covariates on the progression of both (1) torsional deformation over predefined numbers of cycles and (2) number of cycles until predefined torsional deformation levels (*P* ≥ 0.10). Numbers of cycles to failure and corresponding peak torque at failure were significantly higher for group 2 (7532 ± 786 and 16.06 ± 1.57 Nm) versus group 1 (5650 ± 1159 and 12.3 ± 2.32 Nm), respectively (*P* = 0.01) (Figure 5).

**Figure 6:**
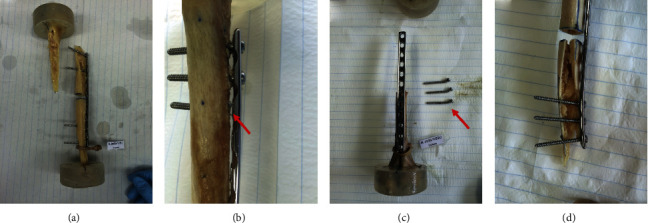
Photographs of exemplified specimens presenting four different catastrophic failure types identified in the current study: (a) bone fracture through the proximal screws without any screw pullout; (b) breakage of proximal screw #3 and simultaneous bending and pullout of screws #1 and #2 (the latter in group 2 only) without any bone fracture; (c) bending and pullout of the proximal screws, followed by a bone fracture through these screws; (d) bone fracture through the three distal screws without any previous screw pullout or bending (in group 2 only).

Four different catastrophic failure types were identified as illustrated in Figure 6. Failure type 1 (group 1: 3 cases; group 2: 2 cases) was defined as a bone fracture through the proximal screws without any screw pullout, screw bending, or screw breakage (Figure 6(a)). Failure type 2 (group 1: 3 cases; group 2: 2 cases) was defined as breakage of the proximal screw #3 and simultaneous bending and pullout of screws #1 and #2 (the latter only in the group 2 with 3 screws per fragment), without any bone fracture (Figure 6(b)). Failure type 3, defined as bending and pullout of the proximal 3 screws, followed by a bone fracture through these screws, occurred in group 2 only (1 case, Figure 6(c)). Failure type 4, characterized as a bone fracture through the 3 distal screws without any previous screw pullout or bending, occurred in group 2 only (1 case, Figure 6(d)).

## 4. Discussion

This study analyzed the biomechanical performance of comminuted humeral shaft fractures fixed with LC-DCP in bridge plating fashion with the use of either two or three screws per fragment. As hypothesized, no significant differences were detected between the two plate constructs with regard to their primary stability. However, the three-screw configuration proved to provide better stability under dynamic loading. Specifically, the numbers of load cycles until several predefined torsional deformation levels and to construct failure were significantly higher when three screws per fragment were used. Adding a third screw in each fragment can therefore enhance the construct stability under repetitive loading, while not altering the working length of the implant. From a clinical perspective, these results provide evidence that the immediate postoperative care may be accelerated or extended from early passive and active motions to more demanding repetitive daily life activities or exercises against resistance with the aim of shortening the rehabilitation period, although this comes at the expense of a more invasive surgical procedure.

The results of the current study suggest that by adding one screw per fragment, the overall peak bone stress is reduced while being distributed over a larger area, making the bone-screw interface less prone to fracture. The effect of increased surface bone strains in certain patterns of screw omissions was previously demonstrated [31]. Although the authors claimed that this effect may have positive implications in terms of enhanced bone healing by generating an optimized strain environment at the fracture site, their study results emphasize the potential negative impact of such a more compliant construct. From our present point of view, the choice of best possible screw-plate configuration proves once again to be a balancing act between fixation stability and flexibility, which has been a subject of debates for years.

The use of a long narrow dynamic compression plate with two 4.5 mm cortical screws per fragment was initially recommended for fixation of comminuted diaphyseal fractures of the humerus [1, 2]. Meanwhile, the range of applicable implants has been extended to broad plates and LCPs with the use of up to six screws per fragment [2, 18–20]. Construct stiffness was analyzed using various screw configurations in combination with a 12-hole 4.5 mm titanium LCP in uniform composite bone cylinders, and it was reported that the number of screws affected construct stiffness; however, placement of more than three screws per fragment contributed only little to the increase in axial and torsional stiffness [8]. Besides the number of screws, their location also influences the construct stiffness of plated diaphyseal fractures; e.g., greater distance between the two closest screws to the fracture site, i.e., the working length, results in lower axial and torsional stiffness. Previous studies, testing compression plates in artificial bone models and human cadaveric ulnae, demonstrated that another crucial factor for the strength of bone-plate constructs is the distance between the innermost and outermost screws of each fragment [32, 33]. A positive correlation between this distance and the construct strength was reported. However, in our study, the third screw inserted in each fragment neither altered the working length of the plate because of its placement between the first two screws nor influenced the distance between the innermost and outermost screws.

In a previous study featuring the most similar attributes to the present one, the authors compared biomechanically plating of humeral shaft fractures using two- versus three-screw configurations per fragment in paired cadaveric humeri; however, they used LCPs with locking screws [24]. In concordance with our results, they concluded that the addition of a third screw per fragment resulted in no significant increase in initial construct stiffness. However, in contrast to our findings, their results demonstrated lower failure loads with three locking screws in comparison to the two-screw configuration. This provides another evidence that the mechanics of locking plates is different from conventional compression plates and that by increasing the number of cortical screws, the mechanical strength of compression plate constructs can be leveled up to those of locking plates. The strength can be even further increased if a special cross-screw fence-like technique of cortical screw placement is applied [34]. Notwithstanding this, it has to be taken into account that in fracture patterns different from the present one, such as those involving interfragmentary bony support, a different screw configuration, providing less rigid construct fixation, may be mechanically advantageous over the one with all plate holes being occupied. A previous study reported that some more flexible constructs exhibit advantages over stiffer ones in terms of withstanding dynamic loading [35], and its findings were further substantiated by demonstrating that stiffer compression plate constructs failed earlier than more flexible ones with the use of locking plates [34].

To our knowledge, this is the first study exploring the effect of two different screw configurations in plated comminuted humeral shaft fractures under progressively increasing cyclic torsional loading to failure, while keeping a constant working length of the constructs. The presented loading protocol may explain the fact that in the present study, the positive effect of additional screw placement was clearly better demonstrated than that in the previously mentioned work. By combining progressively increasing torsional loading with a sufficiently high number of cycles, the current study is aimed at investigating the accentuated fatigue-like behavior—an approach being found useful in other studies targeting construct failure within a reasonable cycling period [36, 37]. Fatigue performance of constructs under axial loading over a maximum of one million cycles was previously assessed, however rather focusing on the effect of their different gap sizes and working lengths [8]. On the other hand, locked-plated constructs were subjected under torsional loading over 1000 cycles prior to retesting them statically in three different loading modalities [24, 38]. As the authors acknowledged, this number of cycles may have been too low to demonstrate a degrading effect of fatigue-like loading on the change in construct stiffness. A significantly higher cyclic number was applied in another biomechanical study, investigating the evolution of axial stiffness in locked plates with slotted near cortical holes [39].

The present study investigated primary and secondary stability of plated comminuted humeral shaft fractures. The primary stability was evaluated via calculation of the initial construct stiffness under four loading scenarios simulating different arm motions. The constructs using three screws per fragment performed marginally better than those using two screws, with no significant differences identified between them. Moreover, the relative stiffness of both constructs in the different loading modes was comparable with the corresponding theoretically calculated values for an isolated plate, proving that the implant is solely responsible for the load transmission in a fracture configuration without interfragmentary bony support. The normalized stiffness of some bone-implant constructs was considerably below the nominal stiffness of the intact state due to the relatively large plate working length. In some fracture pattern situations, this may result in too flexible constructs and the necessity of their stiffening by decreasing the working length. The design of our study followed explicitly surgical prescriptions in a previous study, where the authors concluded that occupying only the peripheral three plate holes with screws was related to satisfactory fracture healing, whereas the use of more central screws in proximity to the fracture zone was associated with excessive stability and no callus formation [2]. In this regard, our study design seems to be clinically relevant. However, it shall be accentuated that other fracture patterns may require more rigid fixations with shorter working length, where the effect of adding a third screw may not be guaranteed.

The secondary stability in the current study was evaluated by subjecting the specimens to pure cyclic torsional loading to failure, which reflected the fact that the upper extremities are predominantly loaded in torsion [7, 8]. As the stresses acting in the humeral shaft during daily life activities are not reported in the literature up to date, only a comparison between the currently applied torques and the reported measured or calculated glenohumeral joint reaction moments seems practical. The glenohumeral contact moments measured in vivo reached maximum values of 5.2 Nm for arm flexion movements up to 120° [40]. Using an inverse dynamic shoulder model, the calculated net moments acting in the glenohumeral joint were assessed within a range of 4–12 Nm for patients performing 12 different activities of daily living [41]. Furthermore, the Static Strength Prediction Program [42] was applied to measure the glenohumeral net moments in three individuals performing motions in four different planes to find that the values varied between 0 and 13 Nm [43]. Therefore, the currently applied torsional moments up to construct failure are comparable with the reported values, which clearly indicates that the use of three screws per fracture fragment has potential advantageous implications for clinical use.

BMD and cortical thickness of the specimens did not significantly influence the outcomes. This absence of sensitivity and the fact that no screw loosening was observed during cyclic loading imply that compression plating using LC-DCPs may be an amenable and more cost-efficient treatment option for humeral shaft fractures compared to locked plating. A previous study reported significant toggling in artificial humeri fixed with nonlocked constructs [12]. However, these models were explicitly used to mimic osteoporotic bone quality. Furthermore, in another study by the same first author [44] using cadaveric radius models from elderly donors, only subtle biomechanical advantages were confirmed for LCP over LC-DCP constructs in terms of less energy absorption under anterior bending and higher numbers of cycles until failure under torsional loading.

This study had some limitations inherent to all cadaveric studies. The main limitation was the limited number of specimens used, restricting the statistical power, although some significantly different results between the groups were demonstrated. A further limitation was the evaluation of the parameters of interest by using machine data only. Analysis of interfragmentary movements by other means would have revealed more accurate results and delivered more appropriate answers with respect to fracture healing. Furthermore, in this study, pure bone-implant biomechanics was investigated, neglecting any muscle forces affecting construct behavior. We also acknowledge the fact that more biomechanical data could have been collected by periodically interrupting the cyclic torsional tests after predefined cyclic numbers to assess the evolution of screw loosening torques and quasistatically retest the specimens in the different loading modalities. These efforts were abandoned for the sake of maintaining flawless conditions during cyclic testing. Another limitation in our study was related to the simulated working length that may have not reflected the different clinical scenarios but was specifically tailored to the used fracture gap model. Finally, the current study was also limited to the extent that the screw tightening torques were applied manually at the discretion of a surgeon. One single surgeon performed all instrumentations to possibly reduce any further instrumentation-related inconsistencies.

The main advantage of the study was the use of paired specimens, ensuring comparable conditions between the 2 groups. Furthermore, the analyzed parameters were chosen to resemble physiological loading conditions during daily life activities.

## 5. Conclusions

From a biomechanical perspective, no significant superiority is identified in terms of primary stability when using two or three screws per fragment for plating of comminuted humeral shaft fractures. However, a three-screw configuration provides better secondary stability and maintains it with a higher resistance towards loss of reduction under dynamic loading for a comminuted fracture without interfragmentary bony support. Therefore, the use of a third screw may be justified when such better secondary stability is required.

## Figures and Tables

**Table 1:** Parameters of interest in both groups in terms of mean and standard deviation together with corresponding *P* values from the statistical analysis.

Parameter of interest	Group		*P* value
	1	2	
Morphological data			
BMD (mg HA/cm^3^)	193.3 (49.0)	205.3 (33.0)	0.64
Cortical average (mm)	4.35 (1.66)	4.98 (1.15)	0.34
Stiffness (plated)			
Lateral bending (Nm/°)	2.8 (1.4)	3.1 (1.2)	0.68
Anterior bending (Nm/°)	1.4 (0.2)	1.5 (0.2)	0.20
Axial bending (N/mm)	387.5 (143.3)	394.4 (134.3)	0.93
Torsion (Nm/°)	0.7 (0.2)	0.8 (0.1)	0.22
Normalized stiffness (%)			
Lateral bending	85.1 (59.5)	61.5 (18.1)	0.42
Anterior bending	35.8 (9.3)	31.6 (13.2)	0.52
Axial bending	23.1 (7.7)	21.2 (6.3)	0.60
Torsion	23.8 (4.4)	23.0 (1.7)	0.72
Torsional deformation (°)			
500 cycles	0.40 (0.58)	0.15 (0.08)	0.36
1000 cycles	1.51 (1.97)	0.63 (0.47)	0.31
1500 cycles	2.77 (3.27)	1.31 (1.00)	0.30
2000 cycles	4.55 (5.41)	2.04 (1.48)	0.29
2500 cycles	6.35 (7.22)	2.89 (1.99)	0.27
3000 cycles	8.13 (8.70)	3.74 (2.45)	0.24
3500 cycles	10.83 (11.53)	4.69 (2.94)	0.22
Cycles and peak torque at torsional deformation			
5°	3151 (1894) 7.3 (4.8) nm	4240 (1730) 9.5 (4.5) nm	0.24
10°	4122 (1933) 9.2 (4.9) nm	6080 (1862) 13.2 (4.7) nm	0.03
15°	4857 (1824) 10.7 (4.6) nm	6887 (1347) 14.8 (3.7) nm	0.01
20°	5328 (1617) 11.7 (4.2) nm	7393 (945) 15.8 (1.9) nm	0.01
Cycles and peak torque at failure	5650 (1159) 12.3 (2.32) Nm	7532 (786) 16.06 (1.57) Nm	0.01

## Data Availability

The data used to support the findings of this study may be released upon application to the AO Research Institute Davos.
